# Is virtual reality training for lumbosacral freehand pedicle screw placement an alternative to conventional training?

**DOI:** 10.1007/s00590-025-04611-y

**Published:** 2025-12-15

**Authors:** Lukas Urbanschitz, Filippo Mandelli, Cordula Netzer, Philippe Cattin, Stefan Schären

**Affiliations:** 1 Department of Spine Surgery, University Hospital Basel, Basel, Switzerland; 2 Department of Biomedical Engineering, University of Basel, Basel, Switzerland

**Keywords:** Virtual reality, Surgical training, Spine, Lumbar, Pedicle screw

## Abstract

**Purpose:**

The goal of this study was to determine whether residents can be taught to place spinal pedicle screws in a virtual reality (VR) environment and if the results are comparable to conventional training.

**Methods:**

In this pilot study, four neurosurgery residents with less than one year of experience in spine surgery performed three rounds of pedicle screw instrumentation from L1 to S1. Both groups performed the first instrumentation after self-instruction. Before the second instrumentation, one group had conventional training, which was instruction on a synthetic bone model, and the other group was trained in a virtual environment. For the last instrumentation, both groups had both trainings. After completion, screw placement was rated by a blinded reader according to the trajectory and integrity of the screw canal. For statistical analysis, we used logistic regression for individual variability with Firth’s bias reduction method.

**Results:**

After self-instruction 21/48 (44%) screws were misplaced, 6 (25%) in the group before conventional training and 15 (64%) in the group before VR-training. Both trainings led to a significant reduction of screw misplacement, with only a single misplaced screw in the VR-training group (2%, *p* < 0.001) and none in the conventional training group (*p* = 0.008). In both groups, no significant difference was found regarding the misplacement rate from the first to the second training (*p* = 0.19).

**Conclusions:**

Training in a virtual reality environment for lumbosacral pedicle screw placement is associated with an easy learning curve. Comparable outcomes are observed with conventional training, however, definitive conclusions regarding the relative effectiveness of either approach will require validation in studies with larger cohorts.

## Introduction

The placement of pedicle screws in the lumbosacral spine is one of the staple skills needed in spine surgery. Nowadays, several technical aids are available to increase the accuracy of the placement of these screws [1–3]. Lumbar navigation has proven to decrease the chance of implant misplacement, especially in complex spinal deformity and revision cases [4]. Also, in 2016, the first study about the placement of pedicle screws using augmented reality was published [5]. Despite the presence of these technical auxiliaries, they may not be a replacement for proper surgical training. All of these devices may be defective at some point and also have their pitfalls, such as rotational and translational error, increasing inaccuracy with distance from the reference and prolonged operating time [6–9].

With the availability of high-power graphics cards, virtual reality (VR) glasses have become widely available. VR- training offers several advantages over conventional training courses, as one can enter a virtual environment regardless of travel restrictions or costs. This may bring surgical education to even remote areas and can also save a lot of time spent on travel. However, whether surgical training, namely the placement of lumbosacral pedicle screws, is feasible in a virtual training environment is still not defined. Therefore, it was the goal of this study to determine whether residents can be taught to place spinal pedicle screws in a virtual reality environment and if the results are comparable to conventional training.

## Materials and methods

### Hypothesis

Residents can be trained to place lumbosacral pedicle screws using virtual reality, with a learning curve comparable to that of conventional training.

## Study set-up

As this study did not involve either sensitive patient data or human specimens, according to local ethics regulations, approval of the local ethics committee was not necessary for the conduction of this study.

**Fig. 1:**
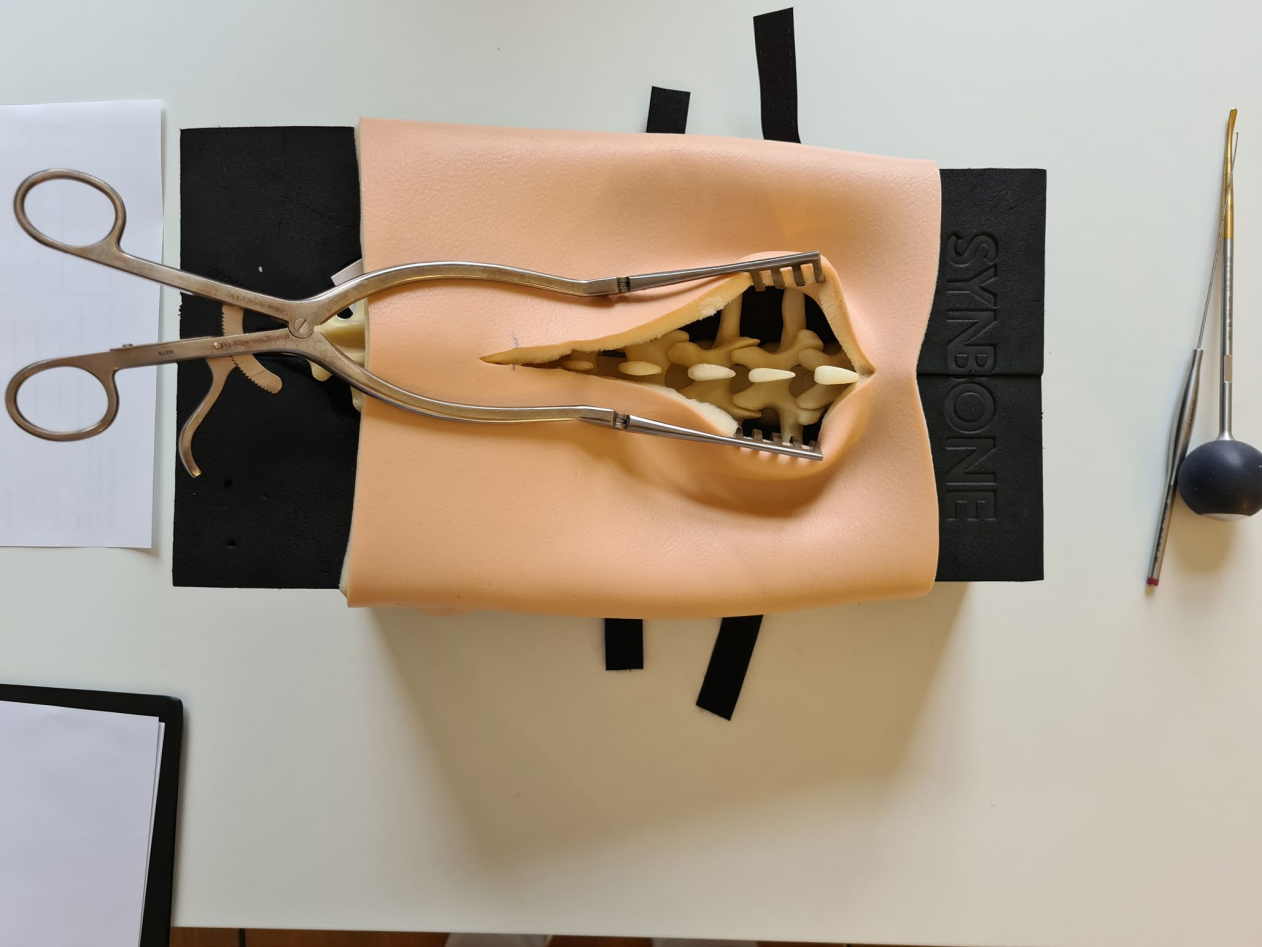
Shows the setup of the training provided to the participants. The sawbone was embedded in a spine bed and covered with an artificial skin flap. For the exposure, every participant had a surgical wound retractor available

Four medical school graduates with less than one year of experience in spinal surgery were enrolled in this study. None of the participants was previously trained in pedicle screw placement. Two female and two male participants were randomized into two groups with an equal gender distribution. Both groups started with self-instruction according to the AO Surgery reference instructions for lumbar and sacral pedicle screw placement (https://surgeryreference.aofoundation.org/). After self-instruction, both groups instrumented a lumbosacral sawbone from L1 to S1 in that order (Synbone Spine lumbar L1–L5 Sacrum; LD9379). The bone models were embedded in spine-beds and covered with an artificial skin flap (Fig. 1).

**Fig. 2:**
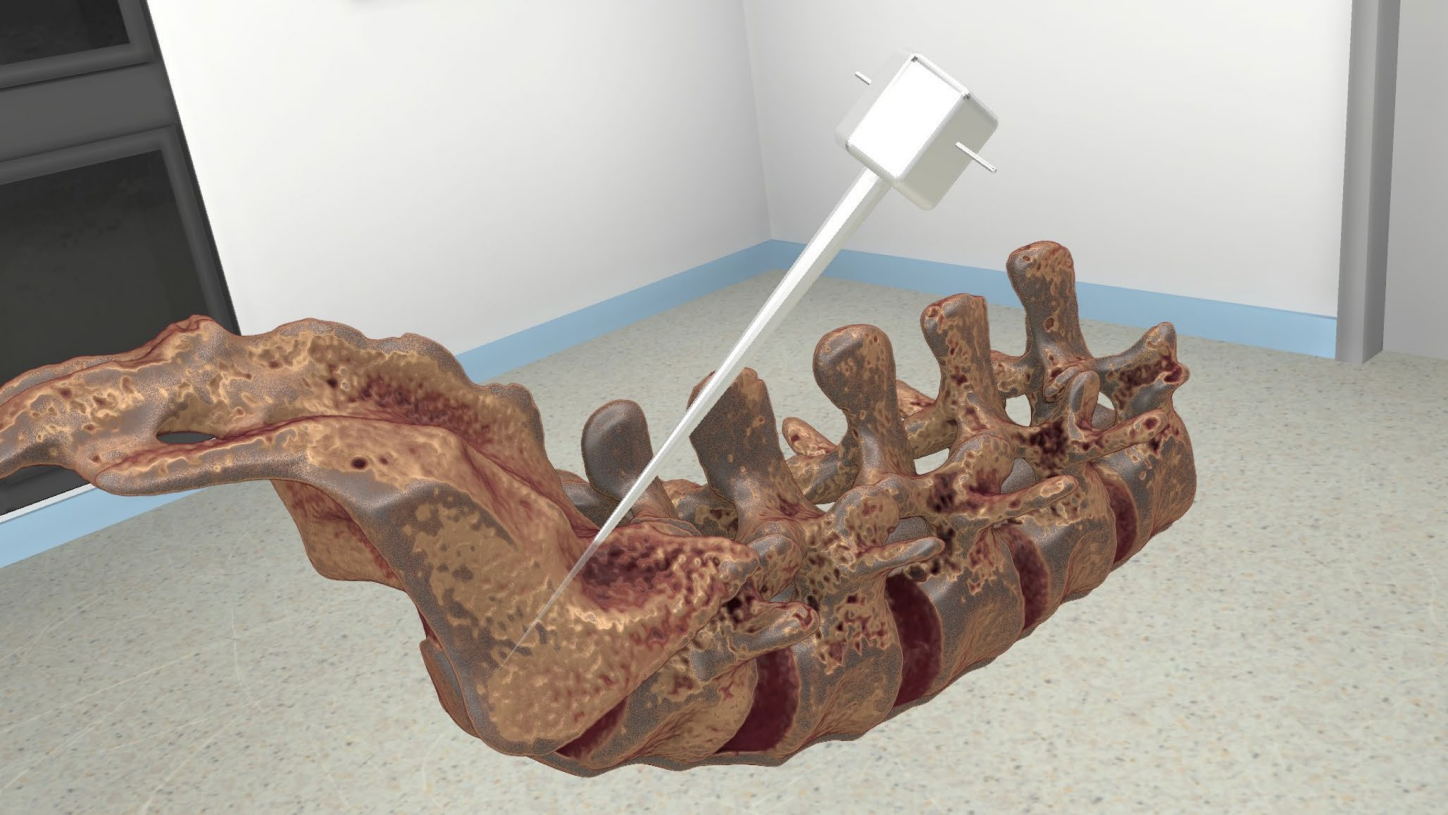
Shows the 3-dimensional reconstruction of a previously acquired computed tomography of the sawbone. For instructional purposes, a 3-dimensional pointer was added to simulate the screw trajectory

Instrument- and implant use were instructed by a company representative (EXPEDIUM^®^ 5.5 System, DePuy Synthes, Johnson and Johnson). The provided implants consisted of screws with a length of 45 mm and a diameter of 6 mm. After completion of the screw placement, all were removed for the screw canal evaluation. Before the second round of instrumentation, both groups received training. Group A had face-to-face training on a sawbone model, which is usually the case at instructional courses and will, therefore, be referenced to as conventional training. Group B was trained according to the same principles in a VR environment (Specto 5.0.0. of Specto Medical^®^) and a 3 dimensionally reconstructed model of a CT-Scan of the Sawbone model (Fig. 2) using the HP G2 Virtual Reality glasses.

**Fig. 3:**
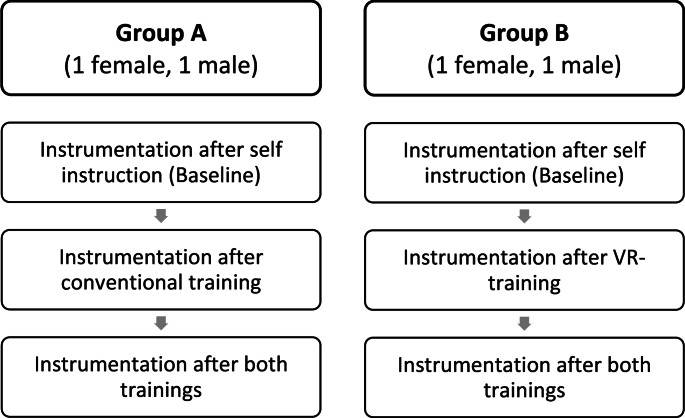
Shows the group allocation and the training course that every participant underwent. Group A underwent conventional training first, whilst Group B underwent VR-training. Before the third instrumentation, Group A had VR-training and Group B vice versa

After the instruction, all participants instrumented their second sawbone model. Before the instrumentation of the third sawbone, every participant received the training they did not have before. Therefore, the conventionally trained group was trained in the virtual space and vice versa (Fig. 3).

After completion of the instrumentation, the screw placement on all sawbones was rated according to the trajectory and integrity of the screw canal by a blinded reader. For blinding, all sawbones were marked with letters and randomly assigned during the instrumentation process. The assignment of the sawbones was documented anonymously and no access to this information was granted to the rater, who was an experienced spine surgeon.

## Rating of screw placement

The rater received all the sawbones after the removal of the screws. Only screws that were placed entirely intrapedicular and inside the vertebral body were graded as correct. Any osseous cortical breach, regardless of wether anterior to the vertebral body, into the disc space, laterally, cranial or inferior, was rated as misplaced. The screw canal was palpated with a bone probe to ensure intradiscal placement was not missed. For S1, if both screws were convergent, an anterior cortical breach of the S1 vertebra was still graded as correct.

### Statistical analysis

Values are provided in absolute numbers and in percentages. To assess the differences in successful screw placements between trainings, we used logistic regression with person as an added confounder. Because of sparseness and quasicomplete separation of successful screw placements, we used Firth’s bias reduction method to reduce bias of maximum likelihood estimates. A* p*-value of less than 0.05 was considered significant. The analysis was performed using the R package “logistf” version 1.26.0.

## Results

A total of 144 pedicle screws were placed free hand, of which 48 were placed after self-instruction, 48 after the first training (24 after conventional instruction, 24 after VR instruction), and 48 after the second training (both virtual and conventional instruction).

After self-instruction, 21 (44%) screws were misplaced, 6 (25%) in the group that was going to have conventional training (Group A), and 15 (64%) in the group that was later on taught in VR (Group B). Both trainings led to a significant reduction of screw misplacement, with only a single misplaced screw in the VR-training group B (2%, *p* < 0.001, OR: 0.026, 95% CI 0.002–0.154) and none in the conventional training group A (*p* = 0.008, OR: 0.0581, 95% CI 0.0004–0.546). No difference was found between the groups after the first training (*p* = 0.679, OR: 0.452, 95% CI 0.015–77.7).

After the VR training of the conventionally trained group (Group A), both participants were again able to adequately place every screw. In the group undergoing VR training first and conventional training second (Group B), in the last round of the instrumentation exercise, four screws were found to be misplaced (8%). In both groups, no significant difference was found regarding the misplacement rate from the first to the second training (*p* = 0.19, OR: 3.31, 95% CI 0.568–34.5). A comprehensive table displaying the mode of failure can be found in Table 1. Implant misplacement in all rounds combined was most commonly found at the level S1, as 38% (9) of all screws were misplaced. Three screws at the level L1 (13%), five screws at the level L2 (21%), three screws at the level L3 (13%), two screws at the level L4 (8%) and four screws at the level L5 (17%) were found to be misplaced.

**Table 1:** This table provides the data on screw placement. Every screw that was rated as misplaced is provided with the type of inaccuracy. Group A received conventional training and group B was taught in virtual reality first

	Side	Level	Self-instruction	Comment	First training	Comment	Both trainings	Comment
Male group A	r	L1	Correct		Correct		Correct	
	r	L2	Misplaced	Lateral pedicle breach	Correct		Correct	
	r	L3	Correct		Correct		Correct	
	r	L4	Correct		Correct		Correct	
	r	L5	Correct		Correct		Correct	
	r	S1	Correct		Correct	Bicortical	Correct	
	l	L1	Correct		Correct		Correct	
	l	L2	Misplaced	Lateral pedicle breach	Correct		Correct	
	l	L3	Misplaced	Lateral pedicle breach	Correct		Correct	
	l	L4	Correct		Correct		Correct	
	l	L5	Correct		Correct		Correct	
	l	S1	Misplaced	Cranial disc	Correct		Correct	
Female group A	r	L1	Correct		Correct		Correct	
	r	L2	Correct		Correct		Correct	
	r	L3	Correct		Correct		Correct	
	r	L4	Correct		Correct		Correct	
	r	L5	Correct		Correct		Correct	
	r	S1	Misplaced	Cranial disc	Correct		Correct	
	l	L1	Correct		Correct		Correct	
	l	L2	Correct		Correct		Correct	
	l	L3	Correct		Correct		Correct	
	l	L4	Correct		Correct		Correct	
	l	L5	Correct		Correct		Correct	
	l	S1	Misplaced	Cranial disc	Correct		Correct	
Male group B	r	L1	Misplaced	Cranial disc	Correct		Correct	
	r	L2	Misplaced	Cranial disc	Correct		Correct	
	r	L3	Misplaced	Cranial disc	Correct		Correct	
	r	L4	Misplaced	Cranial disc	Correct		Correct	
	r	L5	Misplaced	Cranial disc	Correct		Misplaced	Cranial disc
	r	S1	Misplaced	Cranial disc	Correct		Correct	
	l	L1	Misplaced	Lateral pedicle breach	Correct		Correct	
	l	L2	Misplaced	Medial pedicle breach	Correct		Correct	
	l	L3	Misplaced	Cranial disc	Correct		Correct	
	l	L4	Correct		Correct		Misplaced	Inferior pedicle breach
	l	L5	Misplaced	Lateral pedicle breach	Correct		Correct	
	l	S1	Misplaced	Cranial disc	Correct	Bicortical	Correct	Bicortical
Female group B	r	L1	Misplaced	Cranial pedicle breach	Correct		Correct	
	r	L2	Correct		Correct		Correct	
	r	L3	Correct		Correct		Correct	
	r	L4	Correct		Correct		Correct	
	r	L5	Misplaced	Cranial disc	Correct		Correct	
	r	S1	Misplaced	Divergent bicortical	Misplaced	Cranial disc	Misplaced	Cranial disc
	l	L1	Correct		Correct		Correct	
	l	L2	Correct		Correct		Misplaced	Inferior pedicle breach
	l	L3	Correct		Correct		Correct	
	l	L4	Correct		Correct		Correct	
	l	L5	Correct		Correct		Correct	
	l	S1	Misplaced	Divergent bicortical	Correct		Correct	

## Discussion

We hypothesized that Training in VR is feasible for lumbosacral pedicle screw placement and that the results are comparable to the current gold standard, instruction on sawbones. According to the data presented in this study, we showed that both VR training and conventional training lead to an easy learning curve in a cohort of aspiring surgeons with less than one year of experience. After a single training, both groups were able to place lumbosacral pedicle screws with a high degree of precision. Both training methods produced comparable results, with no significant differences observed in performance metrics. Also, we found no further improvement in pedicle screw placement after the second training.

In remote areas, surgeons may not have access to proper training and may have to rely on self-instruction and the help of a company representative. However, data from this study suggests that self-instruction for pedicle screw placement alone will lead to a high rate of implant misplacement, even if a company representative is available to instruct the handling of the instruments. Therefore, if a surgeon has insufficient training in pedicle screw placement, self-instruction may not be an option, as pedicle screw placement is a complex task for which adequate training and supervision are paramount. VR training may fill the gap wherever conventional training is not available.

So far, VR has been mostly used as an adjunct for surgical training with haptic devices and for surgical planning [10]. Only a few studies are focusing on spinal lumbar instrumentation training using VR as a tool for the instruction of novice surgeons. Xin et al. used an immersive VR surgical simulator and compared its efficiency to conventional training. Further accuracy was determined by fluoroscopy intraoperatively in a real patient [11]. However, unacceptable accuracy was present in > 55% of all cases, even after training [11]. Gasco et al. published a series of pedicle screws placed after augmented reality training with a specialized immersive touch simulator compared to conventional training [12]. They demonstrated that after VR training, participants had over 50% fewer implant placement errors compared to participants with only conventional instruction on a bone model. However, even though the VR-training group had better results, still 25 total errors were made within only 26 screws, even in the better performing VR-training group [12]. The high divergence in numbers of errors compared to those found in this study is explained by the assessment of errors, as other authors also counted marginal errors in trajectory as inaccuracy [11, 12]. Also, length was predetermined in this study and therefore, screw length inaccuracy was not relevant.

The greatest limitation of this study was the low number of participants. Also, there was a severe disparity in baseline performance, especially the male participant in Group B (VR-Training). We aimed to minimize bias from varying levels of individual talent by having each participant perform an instrumentation task using only self-instruction. This approach allowed us to measure each individual’s improvement due to the training itself, rather than being influenced by their initial skill level. Also, statistical adjustments were made to address this limitation. The male participant in Group B exhibited a higher rate of mistakes after self-instruction compared to the other participants, however, he improved greatly after the first training in VR with a result comparable to the other participants. We were surprised by the extremely easy learning curve of the participants. After a single training, all participants were able to nearly perfectly instrument L1–S1. The reason for this finding and a limiting factor of this study was the splendid visibility of the sawbone and the given perfect exposure of anatomical landmarks. As there was no soft tissue in situ except for a skin flap and no pelvic bone, there were neither vision restraints nor mechanical restraints like the thoracolumbar fascia or the pelvis. One participant had greater inaccuracy following the second training session, which was likely attributable to fatigue. However, due to the low number of participants, a definitive conclusion regarding the relative effectiveness of either approach cannot be drawn and will require validation in studies with larger cohorts. Another limitation of sawbone is the difference in its haptic properties compared to real human bone [13]. However, sawbone offers great reproducibility, as every model is consistently equal to the other one without anatomical differences. Further, synthetic bone has previously been successfully used in the training of spinal surgeons [14, 15]. Also, Gasco et al. provided a setup of a sawbone without a skin flap. Despite one would expect better screw accuracy with better visibility, this was not the case [12].

The reason we chose pedicle screw placement for the validation of VR training is that it resembles a common task chosen when it comes to validation of training, as it offers good reproducibility and the process itself is highly standardized. However, several authors are reporting the successful usage of VR for different tasks, such as transforaminal percutaneous discectomy [16], balloon kyphoplasty [17] or the lateral access to the lumbar spine [18].

There are still many possible applications of VR in spine surgery, especially as technology in this field progresses rapidly. So, even if there is still a long road ahead, it is only a matter of time before it is possible to create a simulation, even for entire complex surgeries. Until then, at least partial sections of surgical procedures may be taught in a virtual training environment.

In this study, VR training has proven to increase the accuracy of pedicle screw placement of the lumbosacral spine. This may offer the opportunity to provide courses in the virtual space, as well as to offer education to remote regions without access to appropriate regional training programs. Creating a virtual training environment may result in better accessibility of surgical education. It may secure the continuation of surgical training programs even in times of travel restrictions due to war or pandemics.

## Conclusion

Pedicle screw placement is a complex task and self-instruction, without proper training, will result in a high rate of implant misplacement. Therefore, training by a skilled instructor is paramount to acquire the skill of pedicle screw placement. Training in a virtual reality environment for lumbosacral pedicle screw placement is associated with an easy learning curve. Comparable outcomes are observed with conventional training, however, a definitive conclusion regarding the relative effectiveness of either approach will require validation in studies with larger cohorts.

## Data Availability

No datasets were generated or analysed during the current study.
